# Supplementary material for: Osteoporosis Associated with Breast Cancer Treatments Based on Types of Hormonal Therapy: A Cross-Sectional Study Using Korean National Sample Data
Source: Medicina (Kaunas). 2023 Aug 22;59(9):1505. doi: 10.3390/medicina59091505 (PMC10532808; doi:10.3390/medicina59091505)

Table S1. Classification of medicine

Category	Anatomical Therapeutic Chemical code
**Alimentary tract**	A02A, A02B, A03, A03A, A03B, A03C, A03F, A03FA A04A, A04A, A05A, A05A, A05B, A06A, A07B, A07C, A07D, A07E, A07F, A07X, A09A, A16A
**Anti-inflammatory and analgesics**	
NSAIDs	M01A, N02AF, N02AX, N02BA
Opioids	N02AA, N02AB, N02AJ
Others	M01CC, N02BE, N02CA, N02CC
**Diagnostics**	V04C, V08A, V08B, V08C, V09, V10BX, V10XA
**Sex hormones**	G02A, G02B, G02C, G03C, G03D, G03F, G03G, G03H, G03XA, G03XB
**Anti-invectives**	A07(A07AA, A07AX, A07BA, A07BB, A07BC, A07CA, A07DA, A07E, A07EA, A07EC, A07FA, A07XA), D01(D01A, D01AC, D01AE, D01BA), G01(G01AA, G01AF, G01AX), J01(J01AA, J01BA, J01CA, J01CE, J01CF, J01CR, J01DB, J01DC, J01DD, J01DE, J01DF, J01DH, J01EE, J01FA, J01FF, J01GB, J01MA, J01RA, J01XA, J01XB, J01XC, J01XD, J01XX), J02(J02AA, J02AB, J02AC, J02AX), J04(J04AB, J04AC, J04AD, J04AK, J04AM, J04BA), J05(J05AB, J05AF, J05AH, J05AJ, J05AP, J05AR, J05AX), J06(J06AA, J06BA, J06BB)
**Other hormones**	H01(H01AA, H01AC, H01BA, H01BB, H01CB, H01CC), H02(H02AA, H02AB, H02BX), H03(H03AA, H03BA, H03BB), H04AA, H05(H05AA, H05BA, H05BX)
**Antineoplastic**	
Selective estrogen receptor modulators	L02BA, G03XC
Aromatase inhibitors	L02BG
Cytotoxic anticancer	L01(L01AA, L01AB, L01AC, L01AX, L01BA, L01BB, L01BC, L01CA, L01CB, L01CD, L01CE, L01DB, L01DC)
Other anticancer	L01(L01XA, L01XC, L01XF, L01XG, L01XK, L01XX), L02(L02AB, L02AE)
Targeted anticancer	L01(L01EA, L01EB, L01EC, L01ED, L01EE, L01EF, L01EG, L01EH, L01EJ, L01EX, L01FA, L01FD, L01FF, L01FG)
**Treatment of bone diseases**	A12AA, A12AX, M05BA, M05BB, M05BX
**Others**	
Neuropsychiatric drugs	N01A, N01B, N03A, N04A, N04B, N05A, N05B, N05C, N06A, N06B, N06D, N07A, N07B, N07C, N07XX, R02AD
**Otorhinolaryngology drugs**	A01(A01A, A01AB, A01AC, A01AD), R01(R01AB, R01AC, R01AD, R01AX, R01BA), R02AA, R03(R03AA, R03AC, R03AK, R03AL, R03BA, R03BB, R03CA, R03CB, R03CC, R03CK, R03DA, R03DC, R03DX), R05(R05, R05CA, R05CB, R05DA, R05DB, R05FA), R06(R06AA, R06AB, R06AD, R06AE, R06AX), R07(R07AB, R07AX), S01(S01A, S01AA, S01AD, S01AE, S01B, S01BA, S01BC, S01CA, S01EA, S01EB, S01EC, S01ED, S01EE, S01FA, S01FB, S01G, S01GX, S01HA, S01JA, S01KA, S01KX, S01L, S01LA, S01XA), S02(S02AA, S02CA), S03AA
**Musculoskeletal disorder drugs**	M02(M02AA, M02AB), M03(M03AB, M03AC, M03AX, M03BA, M03BC, M03BX, M03CA), M09(M09AB, M09AX)
**Cardiovascular and metabolism-related drugs**	A10(A10AB, A10AC, A10AD, A10AE, A10BA, A10BB, A10BD, A10BF, A10BG, A10BH, A10BJ, A10BK, A10BX), B01(B01AA, B01AB, B01AC, B01AD, B01AE, B01AF, B01AX), B02(B02AA, B02AB, B02BA, B02BB, B02BC, B02BD, B02BX), C01(C01AA, C01BC, C01BD, C01BG, C01CA, C01CE, C01DA, C01DX, C01EA, C01EB), C02(C02CA, C02DB, C02DC, C02DD), C03(C03AA, C03BA, C03CA, C03DA, C03DB, C03EA, C03XA), C04(C04AC, C04AD, C04AE, C04AF, C04AX), C05(C05BX, C05CA,

	C05CX), C07(C07AA, C07AB, C07AG, C07BB, C07CB), C08(C08CA, C08DA, C08DB), C09(C09AA, C09BA, C09BB, C09CA, C09DA, C09DB, C09DX), C10(C10AA, C10AB, C10AC, C10AD, C10AX, C10BA, C10BX)
**Blood substitutes and nutritional fluids**	B05(B05AA, B05BA, B05BB, B05BC, B05CA, B05CB, B05CX, B05DB, B05XA, B05XB, B05XC, B05Z, B05ZA, B05ZB), N02AE, V06(V06B, V06DB, V06DX)
**Mineral drugs**	A11(A11BA, A11CC, A11DA, A11EA, A11EB, A11GA, A11HA, A12BA, A12CC), B03(B03A, B03AA, B03AB, B03AC, B03AD, B03BA, B03BB)
**Others**	A14AA, B03XA, C05(C05A, C05AD), D02AE, D03(D03, D03AX), D04AX, D05(D05AX, D05BB), D06(D06AX, D06BA, D06BB, D06BX), D07(D07AA, D07AB, D07AC, D07AD, D07CC, D07CD), D08(D08AC, D08AE, D08AG), D10BA, D11(D11AH, D11AX), G04(G04BD, G04BX, G04CA, G04CB, G04CX), M04(M04AA, M04AB, M04AC), P01(P01AB, P01BA, P01BC), P02(P02BA, P02CA), P03(P03AB, P03AX), V03(V03AB, V03AC, V03AE, V03AF), V07AB
**Immune-related drugs**	L03(L03AA, L03AB, L03AX), L04(L04AA, L04AB, L04AC, L04AD, L04AX)

NSAID: non-steroidal anti-inflammatory drug.

Table S2. Annual average KRW-USD exchange rate and healthcare price index

Year	KRW/USD	Healthcare & medical service price index
2010	1156	0.9164
2011	1107.99	0.9325
2012	1126.76	0.9410
2013	1095.04	0.9444
2014	1053.12	0.9510
2015	1131.52	0.9629
2016	1160.41	0.9725
2017	1130.48	0.9810
2018	1100.58	0.9805
2019	1166.11	0.9851
2020	1180.01	1.0000

Notes: This information is available on the Korean Statistical Information Service website (http://kosis.kr), which represents the relative price level of cost adjusted as of 2020.

Table S3. Annual number of patients, total expense, and per-patient expense of the four groups

Group	Category	Year										
		2010	2011	2012	2013	2014	2015	2016	2017	2018	2019	CAGR
Non-OSP (<55 yrs)	No. of patients	1030	1078	1152	1166	1264	1335	1395	1558	1580	1684	5.61%
	Total costs	$ 3,741,504	$ 4,086,845	$ 4,371,550	$ 4,164,352	$ 5,063,634	$ 4,752,334	$ 5,235,393	$ 6,325,486	$ 6,831,743	$ 6,554,873	6.43%
	Cost per patient	$ 3,633	$ 3,791	$ 3,795	$ 3,571	$ 4,006	$ 3,560	$ 3,753	$ 4,060	$ 4,324	$ 3,892	0.77%
OSP (<55 yrs)	No. of patients	283	317	299	297	298	304	278	343	405	368	2.96%
	Total costs	$ 1,254,447	$ 1,302,249	$ 1,336,033	$ 1,342,755	$ 1,652,247	$ 1,405,990	$ 1,330,013	$ 2,238,059	$ 2,351,352	$ 2,184,712	6.36%
	Cost per patient	$ 4,433	$ 4,108	$ 4,468	$ 4,521	$ 5,544	$ 4,625	$ 4,784	$ 6,525	$ 5,806	$ 5,937	3.30%
Non-OSP (≥55 yrs)	No. of patients	525	501	579	677	703	859	926	1081	1160	1332	10.90%
	Total costs	$ 1,267,995	$ 1,543,795	$ 1,467,073	$ 1,960,327	$ 2,228,401	$ 2,439,288	$ 2,777,788	$ 3,479,668	$ 3,529,545	$ 4,483,592	15.07%
	Cost per patient	$ 2,415	$ 3,081	$ 2,534	$ 2,896	$ 3,170	$ 2,840	$ 3,000	$ 3,219	$ 3,043	$ 3,366	3.76%
OSP (≥55 yrs)	No. of patients	317	309	335	419	463	508	571	638	722	799	10.82%
	Total costs	$ 894,246	$ 984,178	$ 911,967	$ 1,421,879	$ 1,567,482	$ 1,249,938	$ 1,610,457	$ 1,838,536	$ 2,307,589	$ 2,997,587	14.38%
	Cost per patient	$ 2,821	$ 3,185	$ 2,722	$ 3,394	$ 3,385	$ 2,461	$ 2,820	$ 2,882	$ 3,196	$ 3,752	3.22%

OSP: osteoporosis; CAGR: compound annual growth rate. All costs were converted with the annual average exchange rate (KRW/USD, see Supplementary Table 6).

Table S4. Basic characteristics of medical usage

Category	Total		Non-OSP		OSP	
	No. of claims	%	No. of claims	%	No. of claims	%
**Type of visit**						
Outpatient	330,224	91.84	232,713	91.95	97,511	91.56
Inpatient	29,352	8.16	20,361	8.05	8,991	8.44
**Medical institution**						
Tertiary hospital	227,835	63.36	162,478	64.20	65,357	61.37
General hospital	89,325	24.84	61,183	24.18	28,142	26.42
Hospital	8,612	2.40	6,033	2.38	2,579	2.42
Long-term care hospital	8,479	2.36	5,805	2.29	2,674	2.51
Clinic	14,571	4.05	9,964	3.94	4,607	4.33
Oriental medicine hospital	2,424	0.67	1,568	0.62	856	0.80
Oriental medicine clinic	8,330	2.32	6,043	2.39	2,287	2.15

OSP: osteoporosis.

Table S5. Basic characteristics of the patients in subgroups

Category	Non-OSP (<55 yrs)		OSP (<55 yrs)		Non-OSP (≥55 yrs)		OSP (≥55 yrs)		P-value*
	No. of patients	%	No. of patients	%	No. of patients	%	No. of patients	%	
**Age (years)**									<.0.001
< 35	563	4.25	85	2.66	-	-	-	-	
35–44	4,001	30.21	672	21.05	-	-	-	-	
45–54	8,678	65.53	2,435	76.28	-	-	-	-	
55–64	-	-	-	-	5,622	67.39	2,822	55.54	
65–74	-	-	-	-	2,020	24.21	1,699	33.44	
≥ 75	-	-	-	-	701	8.40	560	11.02	
**Sex**									<.0.001
Male	37	0.28	1	0.03	70	0.84	12	0.24	
Female	13,205	99.72	3,191	99.97	8,273	99.16	5,069	99.76	
**Anticancer**									
SERMs	5,884	44.43	1,331	41.70	919	11.02	815	16.04	<.0.001
Aromatase Inhibitors	1,027	7.76	725	22.71	2,358	28.26	2,111	41.55	<.0.001
Cytotoxic anticancer	2,186	16.51	626	19.61	1,001	12.00	574	11.30	<.0.001
Targeted anticancer	747	5.64	255	7.99	464	5.56	269	5.29	<.0.001
Others	1,076	8.13	353	11.06	47	0.56	26	0.51	<.0.001
**Payer type**									<.0.001
NHI	12,760	96.36	3,045	95.39	7,896	94.64	4,729	93.07	
Medicaid	482	3.64	147	4.61	446	5.35	352	6.93	
Others	-	-	-	-	1	0.01	-	-	
**Year**									<.0.001
2010	1,030	7.78	283	8.87	525	6.29	317	6.24	
2011	1,078	8.14	317	9.93	501	6.01	309	6.08	
2012	1,152	8.70	299	9.37	579	6.94	335	6.59	
2013	1,166	8.81	297	9.30	677	8.11	419	8.25	
2014	1,264	9.55	298	9.34	703	8.43	463	9.11	
2015	1,335	10.08	304	9.52	859	10.30	508	10.00	
2016	1,395	10.53	278	8.71	926	11.10	571	11.24	
2017	1,558	11.77	343	10.75	1,081	12.96	638	12.56	
2018	1,580	11.93	405	12.69	1,160	13.90	722	14.21	
2019	1,684	12.72	368	11.53	1,332	15.97	799	15.73	
Compound annual growth rate	5.61%		2.96%		10.90%		10.82%		

NHI: national health insurance; OSP: osteoporosis; SERM: selective estrogen receptor modulators;* Chi-square test

Figure S1. Medical cost of overall drugs**.** A. Non-OSP (<55 years); B. OSP (<55 years); C. Non-OSP (≥55 years); D. OSP (≥50 years); OSP: Osteoporosis.

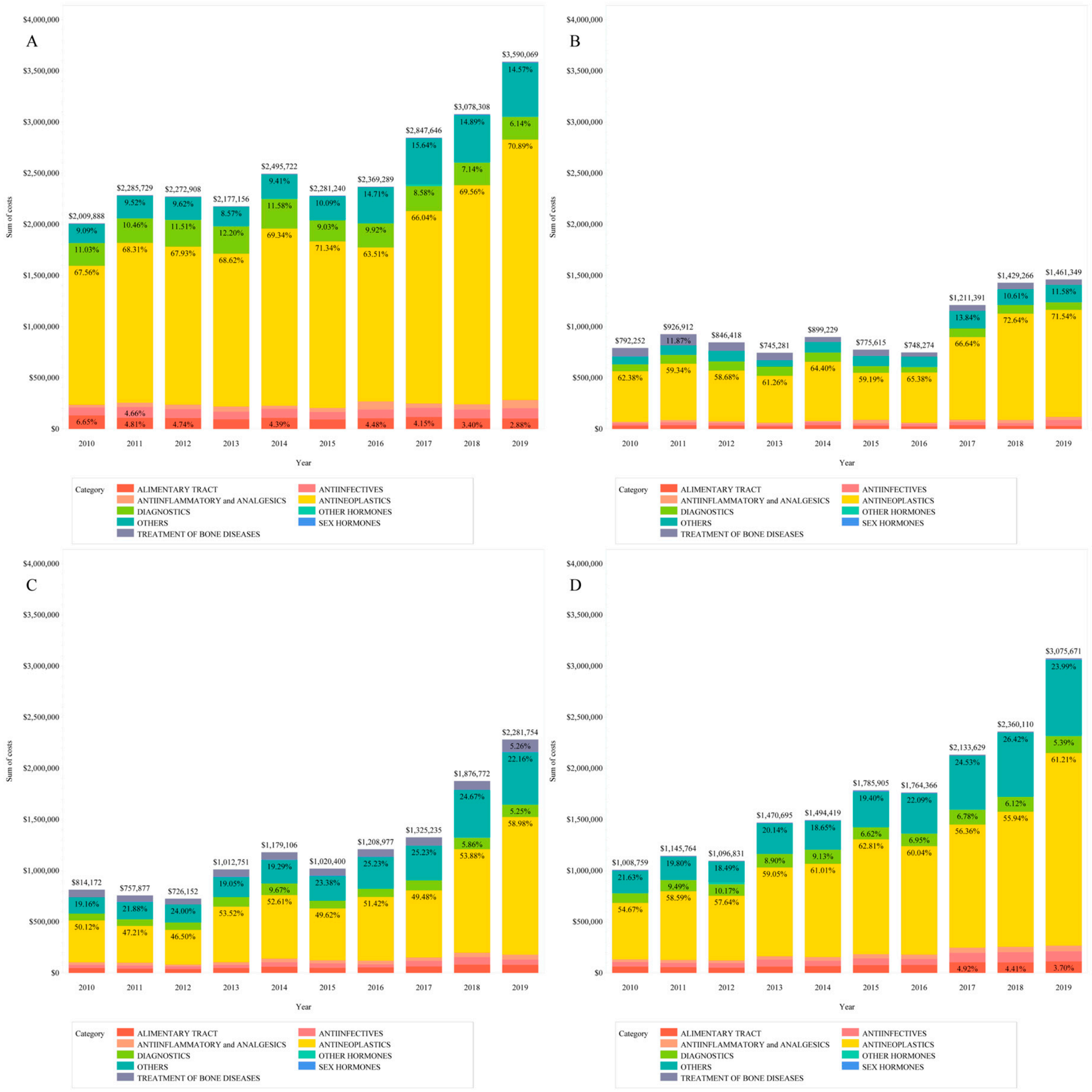

Supplement: Supplementary file 1 [file medicina-59-01505-s001.zip › medicina-2532147-supplementary.pdf]
